# Ubiquitin-conjugating enzyme E2C (UBE2C) is a prognostic indicator for cholangiocarcinoma

**DOI:** 10.1186/s40001-023-01575-9

**Published:** 2023-12-15

**Authors:** Khaa Hoo Ong, Hong-Yue Lai, Ding-Ping Sun, Tzu-Ju Chen, Steven Kuan-Hua Huang, Yu-Feng Tian, Chia-Lin Chou, Yow-Ling Shiue, Ti-Chun Chan, Chien-Feng Li, Yu-Hsuan Kuo

**Affiliations:** 1 Division of Gastroenterology & General Surgery, Department of Surgery, Chi Mei Medical Center, Tainan, 710 Taiwan; 2 Department of Medical Technology, Chung Hwa University of Medical Technology, Tainan, 717 Taiwan; 3 Institute of Biomedical Sciences, National Sun Yat-Sen University, Kaohsiung, 804 Taiwan; 4 Department of Pharmacology, School of Medicine, China Medical University, Taichung, 404333 Taiwan; 5 Department of Clinical Pathology, Chi Mei Medical Center, Tainan, 710 Taiwan; 6 Division of Urology, Department of Surgery, Chi Mei Medical Center, Tainan, 710 Taiwan; 7 Department of Medical Science Industries, College of Health Sciences, Chang Jung Christian University, Tainan, 711 Taiwan; 8 Division of Colon and Rectal Surgery, Department of Surgery, Chi Mei Medical Center, Tainan, 710 Taiwan; 9 Institute of Precision Medicine, National Sun Yat-Sen University, Kaohsiung, 804 Taiwan; 10 Division of Hematology and Oncology, Department of Internal Medicine, Chi-Mei Medical Center, Tainan, 71004 Taiwan; 11 Department of Medical Research, Chi Mei Medical Center, Tainan, 710 Taiwan; 12 National Institute of Cancer Research, National Health Research Institutes, Tainan, 704 Taiwan; 13 Trans-Omic Laboratory for Precision Medicine, Chi Mei Medical Center, Tainan, 710 Taiwan; 14 College of Pharmacy and Science, Chia Nan University, Tainan, 71710 Taiwan

**Keywords:** UBE2C, Prognosis, Cholangiocarcinoma, Ubiquitination

## Abstract

Cholangiocarcinoma is the most common malignant bile duct tumor in Southeast Asia. The special location of cholangiocarcinoma leads to it being difficult to diagnose. Currently, the progress in clinical prognosis outcomes remains abysmal owing to the lack of definitive diagnostic criteria. Therefore, uncovering the potential markers for cholangiocarcinoma is a pressing issue. Ubiquitin-conjugating enzyme E2 C (UBE2C) is a critical ubiquitination enzyme; it is involved in the tumorigenesis of various malignancies and affects the patient’s prognosis. However, there is currently no relevant literature to indicate whether UBE2C is related to the clinical survival outcome of cholangiocarcinoma patients. In this report, we mined the published cholangiocarcinoma transcriptome data set (GSE26566), compared it with the ubiquitination-associated gene (GO:0016567), and identified that UBE2C was highly expressed in cholangiocarcinoma tumor tissue. Moreover, high expression of UBE2C was markedly correlated with surgical margin, primary tumor, histological variants, and histological grade. More specifically, high expression of UBE2C was negatively associated with overall survival, disease-specific survival, local recurrence-free survival, and metastasis-free survival in patients with cholangiocarcinoma. Our findings demonstrate that UBE2C may provide a potential therapeutic marker and prognostic factor for cholangiocarcinoma patients.

**Supplementary Information:**

The online version contains supplementary material available at 10.1186/s40001-023-01575-9.

## Introduction

Cholangiocarcinoma is a relatively rare malignant tumor arising in the bile duct epithelium [1]. Although considered a rare cancer, the incidence of cholangiocarcinoma is on the rise globally, especially in East and Southeast Asia [2]. Because of the lack of symptoms and limitations of diagnostic methods, the 5-year survival rate of cholangiocarcinoma patients is less than 10% [3]. Cholangiocarcinoma is primarily treated through surgery, radiation therapy (RT), and chemotherapy (CT), depending on the disease stage; surgical resection is the primary treatment for early-stage cholangiocarcinoma [4]. However, most patients with cholangiocarcinoma are asymptomatic in the early-stages and are often diagnosed at advanced stages when the tumor has spread to other tissues beyond the bile duct, which dramatically affects treatment outcomes [5]. Clinically, combination therapy with gemcitabine and cisplatin is the primary treatment for advanced-stage cholangiocarcinoma, but only a minority of patients show responses and most patients experience disease relapse after a few weeks or months [6]. Once recurrence or distant metastasis occurs, the 5-year survival rate of patients with cholangiocarcinoma is only about 2% [7, 8]. Therefore, there is an urgent need to identify potential target biomarkers to facilitate the early detection of cholangiocarcinoma.

Ubiquitin-conjugating enzyme E2 C (UBE2C) is a member of the E2 ubiquitin-conjugating enzyme family, and it is the principal regulator of pathways for protein degradation in eukaryotes [9]. Recent studies have reported that UBE2C is also involved in mitotic cyclin disruption, affecting cell cycle progression [10]. Notably, Okamoto Y and colleagues reported that UBE2C RNA and protein expression levels are almost undetectable in normal tissues [11]. In contrast, UBE2C is highly expressed in lung cancer [12], esophageal adenocarcinomas [13], liver cancer [14], nasopharyngeal carcinoma [15], and breast cancer [16], demonstrating that UBE2C may be involved in carcinogenesis and play an essential role in tumorigenesis and cancer progression [17]. For example, inhibition of UBE2C causes G2/M arrest and promotes the apoptosis of melanoma cells [10]. In additionally, knockdown UBE2C expression attenuated cell proliferation and induced cell cycle arrest of S and G2/M phases in nasopharyngeal carcinoma cells [15]. Overexpression of UBE2C enhances cell proliferation, cell migration, and invasion in endometrial cancer [18]. In particular, high expression of UBE2C correlated with an unfavorable prognosis for different cancer types such as breast cancer [19], thyroid carcinoma [20], cervical cancer [21], and gastric cancer [22]. However, the role of UBE2C in intrahepatic cholangiocarcinoma (IHCC) has not yet been thoroughly investigated.

In this study, we mined and analyzed the relationship between the gene associated with ubiquitination (GO:0016567) and tumorigenesis-associated genes in the transcriptome of cholangiocarcinoma (GSE26566). We found that the ubiquitination-associated gene, UBE2C, is most highly expressed in cholangiocarcinoma tumor tissue compared with non-tumor biliary epithelium tissue. Moreover, we further investigated the correlation between UBE2C expression and clinicopathological characterization in cholangiocarcinoma patients. We attempted to clarify the effect of UBE2C expression on the development of cholangiocarcinoma and demonstrated that UBE2C may act as a prognostic factor in cholangiocarcinoma. We found that UBE2C expression was negatively correlated with overall survival, disease-specific survival, local recurrence-free survival, and metastasis-free survival in patients with cholangiocarcinoma. Therefore, these analyses suggest that UBE2C may provide a reliable potential marker for cholangiocarcinoma patients.

## Materials and methods

### Analysis of expression profile from publicly available cholangiocarcinoma transcriptomic dataset

The data sets of mRNA expression came from the GEO database (National Center for Biotechnology Information, USA). We analyzed the data set GSE26566, containing cholangiocarcinoma tumor tissue (*n* = 104) and non-cancerous liver (*n* = 59) samples inspected with Human Genome U133 Plus 2.0 Array from Affymetrix. We calculated the expression level of the gene identified by the probe combination and unnecessary pre-selection or filtrating. Censoring the comparative analysis, the data set was intersected to acquaint the gene, which was expressing significantly. The gene expression of selection on the data set was based on (*p* < 0.0001), and it followed the gene associated with ubiquitination (GO:0016567).

### Patients and tumor specimens

The present study was ratified by the institutional review board of Chi-Mei Medical Center in Taiwan (IRB No. 09912003). The paraffin-embedded tissue blocks were retrieved from 182 intrahepatic cholangiocarcinoma patients. In this study, we included cholangiocarcinoma patients who received curative surgery. The presence of lymph node involvement or distant metastasis was ruled out to guarantee curability. Only individuals with T1-3N0M0 disease were included We gathered patients' retrospective demographic and clinical data, including pathological characteristics, oncological survival follow-up, and cause of mortality. The follow-up time was 3 to 352.7 months, with a mean at 43.4 months (median, 26.7 months). Two pathologists classified tumors through histological subtypes based on WHO classification. The tumor stage was adjusted in the samples using the 7th American Joint Committee on Cancer system.

### Immunohistochemistry (IHC) staining

We used the formalin-fixed, paraffin-embedded (FFPE) cholangiocarcinoma tissue blocks from original histopathological diagnoses of the cases that were subjected to IHC staining. The section of tissue with a thickness of 4 μm was prepared. The sections were de-waxed and then cleared in xylene. Hydration was accomplished by dipping the sections in absolute ethanol. The slides used 3% H2O2 to block endogenous peroxidase and then incubated in the citrate buffer of pH 6.0. The tissue sections were incubated at 4 ℃ overnight with primary antibodies: UBE2C antibody (Clone:23165-31, Abcam, dilution 1:200), following the manufacturers’ recommendations. The secondary antibody reagent HRP polymer was incubated for 30 min at room temperature. After washing the sections and using diaminobenzidine as chromogen, we followed by counterstaining with Mayer’s hematoxylin (Histolab) and were ready for explanation. Two pathologists used the following equation to calculate the H-score to estimate COMP immunoreactivity: H-score = SPi (i + 1), where Pi is the percentage of stained tumor cells in various intensities ranging from 0 to 100%, and I is the degree of staining (0 to 3 +). If there were any scoring disagreements, the two pathologists assessed the slides at the same time and agreed on an H-score. The immunostaining was categorized into low and high expression levels based on the median H-score as previously mentioned [23].

### Gene function prediction

To understand the undisclosed functions of UBE2C in intrahepatic cholangiocarcinoma, we evaluated the relationship between the transcript levels of UBE2C and its co-expressed genes from the cholangiocarcinoma data set (TCGA, Firehose Legacy, *n* = 51). Next, the top 200 differentially expressed genes presenting a positive relationship or a negative relationship to UBE2C were annotated by the Gene Ontology (GO) classification system and were ordered by fold enrichment.

### Statistical analysis

SPSS 14 statistics software was applied for statistical analysis. We compared the clinicopathological parameters and differences between UBE2C expression using the Chi-square test. The endpoints followed overall survival, disease-specific survival, local recurrence-free survival, and metastasis-free survival., following the start date of the radiotherapy to the onset of an event. The Cox proportional hazards model utilized multivariate analysis, and the Kaplan–Meier method compared the survival curves of IHCC patients. All the analyses and *p* < 0.05 were considered to indicate statistical significance.

## Results

### *UBE2C* is significantly up-regulated in cholangiocarcinoma patients

**Fig. 1:**
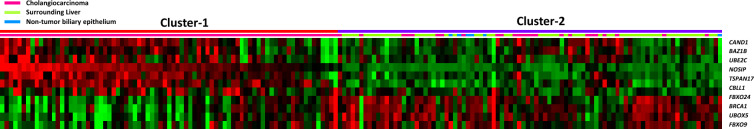
Heatmap showing differential expression of ubiquitination-related genes (GO:0016567) in cholangiocarcinoma tumor tissue and non-tumor bile duct epithelial tissue on the GEO Cholangiocarcinoma database (GSE26566). The mean expression values are black, downregulation is green, and upregulation is red

To evaluate the biomarkers for the diagnosis of cholangiocarcinoma, we mined the cholangiocarcinoma data set (GSE26566) in the Gene Expression Omnibus (GEO) database for analysis of those containing cholangiocarcinoma tumor tissue (*n* = 104) and non-cancerous livers (*n* = 59) and compared them with the gene associated with ubiquitination (GO:0016567) in the Gene Ontology Term (GO Term) database. Heatmap results demonstrated that 11 ubiquitination-associated genes were significantly differentially expressed in the cholangiocarcinoma data set (GSE26566) (Fig. 1). We noted that among these genes, UBE2C (Ubiquitin-conjugating enzyme E2 C) showed the highest fold change in expression between the cholangiocarcinoma tumor tissue and non-cancerous livers (log2 ratio = 1.7925; *p*-value < 0.0001) (Table 1). Therefore, we focused on the ex-pression of UBE2C to explore whether it was associated with the clinicopathologic characterization of cholangiocarcinoma.

**Table 1:** Summary of the alterations of gene associated with ubiquitination (GO:0016567) in cholangiocarcinoma (GSE26566)

Probe	CCA vs non-tumor^#^		CCA vs normal intrahepatic bile duct^&^		Gene symbol	Molecular function	Biological Process
	log ratio	*p*-value	log ratio	*p*-value			
ILMN_1714730	1.7925	< 0.0001	1.845	< 0.0001	*UBE2C*	Ligase activity, ubiquitin-protein ligase activity	Ubiquitin cycle, phosphoinositide-mediated signaling, protein ubiquitination, ubiquitin-dependent protein catabolism, cyclin catabolism, spindle organization and biogenesis, mitosis, cell cycle, cell division
ILMN_1777881	0.8437	0.0117	0.9321	< 0.0001	*TSPAN17*	Ubiquitin-protein ligase activity	Protein ubiquitination
ILMN_1720241	0.6438	0.0032	0.3769	< 0.0001	*TRIP12*	Thyroid hormone receptor binding, ligase activity, ubiquitin-protein ligase activity, binding	Protein ubiquitination
ILMN_1759436	0.5235	0.0132	0.539	< 0.0001	*NOSIP*	Ubiquitin-protein ligase activity	Protein ubiquitination
ILMN_1744647	0.503	0.0215	0.2871	0.0001	*CAND1*	Protein binding	Regulation of transcription; DNA-dependent, protein ubiquitination, transcription, negative regulation of enzyme activity
ILMN_1815475	0.3065	0.0337	0.2021	< 0.0001	*BAZ1B*	Metal ion binding, zinc ion binding, transcription factor activity, protein binding	Regulation of transcription; DNA-dependent, transcription
ILMN_1705433	0.2181	0.0262	0.1568	< 0.0001	*CBLL1*	Ubiquitin-protein ligase activity, zinc ion binding, metal ion binding, nucleic acid binding, protein binding	Negative regulation of cell adhesion, protein ubiquitination, cell–cell adhesion, positive regulation of endocytosis, positive regulation of cell migration
ILMN_1702928	− 0.2834	0.0152	− 0.1773	< 0.0001	*FBXO9*	binding, ubiquitin-protein ligase activity	Protein ubiquitination
ILMN_1686466	− 0.2404	0.0183	− 0.2333	< 0.0001	*FBXO24*	Ubiquitin-protein ligase activity	Protein ubiquitination
ILMN_1666652	− 0.2379	0.0179	− 0.3038	< 0.0001	*BRCA1*	Metal ion binding, transcription coactivator activity, DNA binding, androgen receptor binding, protein binding, ubiquitin-protein ligase activity, zinc ion binding, tubulin binding	Protein ubiquitination, regulation of apoptosis, cell cycle checkpoint, positive regulation of transcription; DNA-dependent, androgen receptor signaling pathway, cell cycle, regulation of transcription from RNA polymerase II promoter, positive regulation of DNA repair, DNA damage response; signal transduction by p53 class mediator resulting in transcription of p21 class mediator, DNA damage response; signal transduction resulting in induction of apoptosis, regulation of transcription from RNA polymerase III promoter, negative regulation of centriole replication, regulation of cell proliferation, DNA repair
ILMN_1652872	− 0.1735	0.0151	− 0.2157	< 0.0001	*UBOX5*	Zinc ion binding, metal ion binding, ubiquitin-protein ligase activity, protein binding	Protein ubiquitination

^**#**^, Comparing cholangiocarcinoma (CCA, *n* = 104) to surrounding liver (*n* = 59) and normal intrahepatic bile duct (*n* = 6); &, Comparing cholangiocarcinoma (CCA, *n* = 104) to normal intrahepatic bile duct (*n* = 6)

### UBECS is associated with poorer clinical pathological parameters of patients with cholangiocarcinoma

**Fig. 2:**
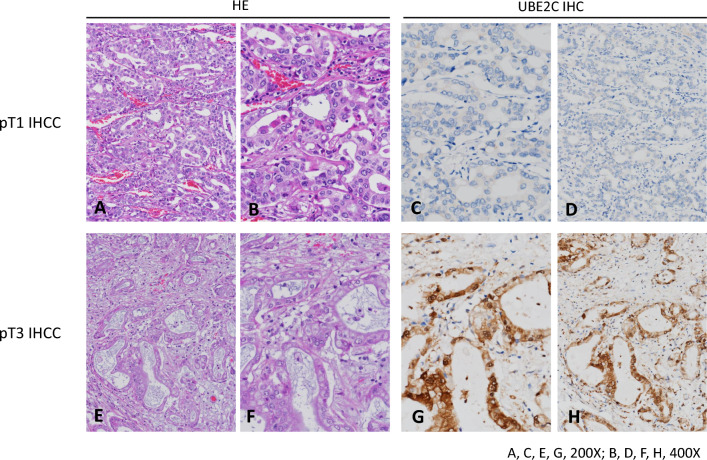
Images of immunohistochemical staining with UBE2C. **A**–**D** Low-stage cholangiocarcinoma tumor tissues have weak UBE2C expression, whereas **E**–**H** the high-stage cholangiocarcinoma tumor tissues have strong UBE2C expression

To determine whether the expression of UBE2C was correlated with the clinicopathologic characteristics in cholangiocarcinoma patients (Table 2), we collected 182 cases of cholangiocarcinoma patients, 108 were male and 74 were female, of which 107 cholangiocarcinoma patients were < 65 years and 75 cholangiocarcinoma patients were male ≥ 65 years. Clinicopathological features analyzed showed that UBE2C-high ex-pression and UBE2C-low expression in cholangiocarcinoma patient tumors were significantly associated with surgical margin (R0 and R1) (*p*-value = 0.029), primary tumor (T1, T2, and T3) (*p*-value = 0.031), histological variant (large duct-type and small duct-type) (*p*-value = 0.001) and histological grade (well-differentiated, moderately differentiated, and poorly differentiated) (*p*-value = 0.023). However, gender, age (< 65 years and ≥ 65 years), hepatitis, and intrahepatic lithiasis showed no significant differences in the tumors of cholangiocarcinoma patients with differential UBE2C expression. Moreover, we determined the expression of UBE2C in human cholangiocarcinoma tumor tissues by immunohistochemical (IHC) staining. We observed that the low-stage cholangiocarcinoma tumor tissues had weak UBE2C expression (Fig. 2A–D), whereas the high-stage cholangiocarcinoma tumor tissues had strong UBE2C expression (Fig. 2E–H). These findings demonstrated that UBE2C was positively correlated with the development of clinicopathological features in cholangiocarcinoma patients, and its expression could be used as a predictor of cholangiocarcinoma.

**Table 2:** Correlations between UBE2C expression and other important clinicopathological parameters in primary localized IHCC

Parameter	Category	Case No	UBE2C Expression		*p*-value
			Low	High	
Gender	Male	108	51	57	0.365
	Female	74	40	37	
Age (years)	< 65	107	51	56	0.451
	≥ 65	75	40	35	
Hepatitis	Hepatitis B	72	42	30	0.191
	Hepatitis C	29	13	16	
	Non-B, non-C	81	36	45	
Intrahepatic lithiasis	Not identified	102	57	65	0.073
	Present	80	34	46	
Surgical margin	R0	163	86	77	0.029*
	R1	19	5	14	
Primary tumor (T)	T1	87	51	36	0.031*
	T2	61	29	32	
	T3	34	11	23	
Histological variants	Large duct type	105	41	64	0.001*
	Small duct type	77	50	27	
Histological Grade	Well differentiated	61	35	26	0.023*
	Moderately differentiated	66	37	29	
	Poorly differentiated	55	19	36	

^*^Statistically significant

### Highly expressed UBE2C is correlated with poor survival outcomes of cholangiocarcinoma patients

**Fig. 3:**
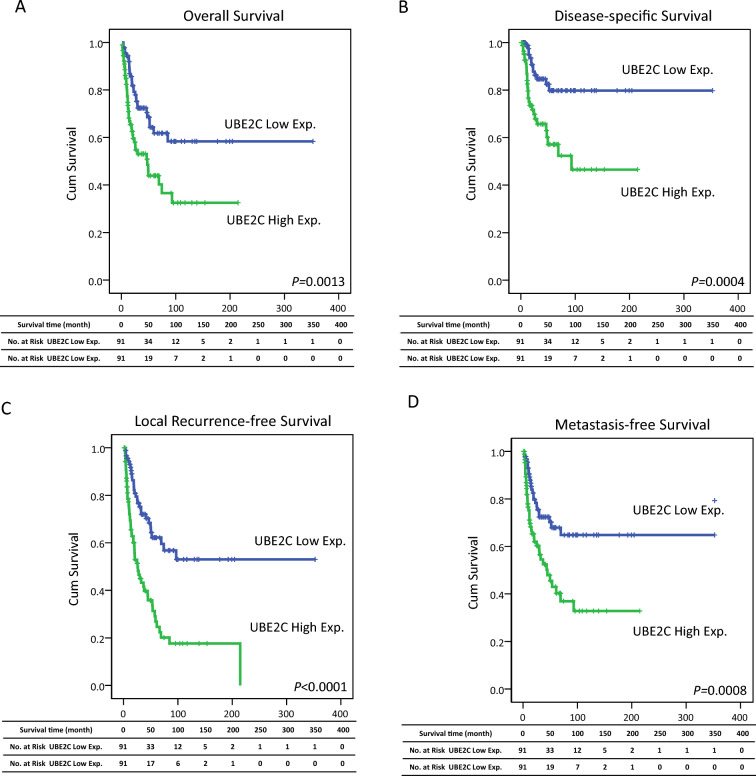
Kaplan–Meier survival curves of cholangiocarcinoma patients with high or low expressions of UBE2C. **A** Overall survival, **B** disease-specific survival, **C** local recurrence-free survival, and **D** metastasis-free survival

To identify whether expression of UBE2C affects survival outcomes in patients with cholangiocarcinoma, Kaplan–Meier analysis indicated that UBE2C high expression was markedly associated with poorer overall survival (Fig. 3A; *p*-value = 0.0013), disease-specific survival (Fig. 3B; *p*-value = 0.0004), local recurrence-free survival (Fig. 3C; *p*-value < 0.0001), and metastasis-free survival (Fig. 3D; *p*-value = 0.0008). Moreover, to further determine the association between the prognostic significance of UBE2C expression and clinicopathological parameters in cholangiocarcinoma patients, we performed univariate and multivariate analyses. The overall survival was markedly correlated with gender (male and female; *p*-value = 0.0254) by univariate analysis, but multivariate analysis showed that gender was not significantly correlated with overall survival. In addition, surgical margin (R0 and R1), primary tumor staging (T1, T2, and T3), and UBE2C expression (high and low expression) were markedly correlated with overall survival and disease-specific survival by the models of univariate and multi-variate analysis. However, the age (< 65 years and ≥ 65 years), hepatitis (hepatitis B, hepatitis C, and non-hepatitis B and non-hepatitis C), intrahepatic lithiasis (not identified and present), histological variants (large duct-type and small duct-type), and histological grade (well, moderately and poorly) were not significantly different in overall survival and disease-specific survival (Table 3). Moreover, we analyzed the correlation of local recurrence-free and metastasis-free survival with clinical characteristics through univariate and multivariate analyses. The results revealed that surgical margin, primary tumor staging, histological variants, and UBE2C expression were significantly correlated with local recurrence-free survival and metastasis-free survival. Among them, the histological grade was significantly associated with local recurrence-free survival (*p*-value = 0.0299) in univariate analyses but not in multivariate analyses (Table 4). Taken together, these analyses indicated that UBE2C might provide a new diagnostic marker for cholangiocarcinoma.

**Table 3:** Univariate log-rank and multivariate analyses for overall and disease-specific survivals in primary localized IHCC

Parameter	Category	Case No	Overall survival					Disease-specific survival				
			Univariate analysis		Multivariate analysis			Univariate analysis		Multivariate analysis		
			No. of event	*p*-value	R.R	95% C.I	*p*-value	No. of event	*p*-value	R.R	95% C.I	p-value
Gender	Male	108	50	0.0254*	1	–	0.105	9	0.0072*	1	–	0.048*
	Female	74	21		1.532	0.914–2.566	–	32		2.118	1.006–4.457	–
Age (years)	< 65	107	37	0.2626	–	–	-	28	0.2125	–	–	–
	≥ 65	75	34		–	–	-	13		–	–	–
Hepatitis	Hepatitis B	72	32	0.2379	–	–	-	16	0.4561	–	–	–
	Hepatitis C	29	8		–	–	–	19		–	–	–
	Non-B, non-C	81	31		–	–	–	6		–	–	–
Intrahepatic lithiasis	Not identified	102	36	0.2831	–	–	–	19	0.1613	–	–	–
	Present	80	35		–	–	–	22		–	–	–
Surgical margin	R0	163	59	< 0.0001*	1	–	0.004*	31	< 0.0001*	1	–	< 0.001*
	R1	19	12		2.756	1.389–5.467		10		4.523	2.020–10.126	
Primary tumor (T)	T1	87	25	0.0001*	1	–	0.020*	9	< 0.0001*	1	–	0.004*
	T2	61	27		1.783	1.029–3.089	–	19		3.521	1.583–7.830	–
	T3	34	19		2.371	1.242–4.524	–	13		3.735	1.519–9.184	–
Histological variants	Large duct type	105	43	0.4281	–	–	–	27	0.1984	–	–	–
	Small duct type	77	28		–	–	–	14		–	–	–
Histological Grade (Differentiation)	Well	61	20	0.1663	–	–	–	12	0.3881	–	–	–
	Moderately	66	28		–	–	–	16		–	–	–
	Poorly	55	23		–	–	–	13		–	–	–
UBE2C Exp	Low expression	91	28	**0.0013**	1	–	0.046*-	13	0.0004*	1	–	0.019*-
	High expression	91	43		1.668	1.068–2.758	–	28		2.289	1.143–4.583	–

^*^ Statistically significant

**Table 4:** Univariate log-rank and multivariate analyses for local recurrence-free and metastasis-free survivals in primary localized IHCC

Parameter	Category	Case No	Local recurrence-free survival					Metastasis-free survival				
			Univariate analysis		Multivariate analysis			Univariate analysis		Multivariate analysis		
			No. of event	*p*-value	R.R	95% C.I	*p*-value	No. of event	*p*-value	R.R	95% C.I	*p*-value
Gender	Male	108	54	0.2170	–	–	–	21	0.1008	–	–	–
	Female	74	31		–	–	–	44		–	–	–
Age (years)	< 65	107	55	0.2993	–	–	–	42	0.2936	–	–	–
	≥ 65	75	30		–	–	–	23		–	–	–
Hepatitis	Hepatitis B	72	33	0.7333	–	–	–	26	0.8762	–	–	–
	Hepatitis C	29	13		–	–	–	11		–	–	–
	Non-B, non-C	81	39		–	–	–	28		–	–	–
Intrahepatic lithiasis	Not identified	102	41	0.0551	–	–	–	31	0.1000	–	–	–
	Present	80	44		–	–	–	34		–	–	–
Surgical margin	R0	163	71	< 0.0001*	1	–	< 0.001*	54	< 0.0001*	1		0.014*
	R1	19	14		3.428	1.764–6.663		11		2.467	1.202–5.062	
Primary tumor (T)	T1	87	28	< 0.0001*	1	-	0.015*	21	< 0.0001*	1	-	0.006*
	T2	61	32		1.715	0.985–2.985		26		2.180	1.220–3.895	
	T3	34	25		2.459	1.327–4.559		18		2.757	1.394–5.454	
Histological variants	Large duct type	105	58	0.0085*	1	–	0.591	43	0.0759	–	–	–
	Small duct type	77	27		0.870	0.523–1.446		22		–	–	–
Histological Grade(Differentiation)	Well	61	28	0.0299*	1	–	0.627	22	0.1794	–	–	–
	Moderately	66	27		0.960	0.551–1.671		22		–	–	–
	Poorly	55	30		1.238	0.719–2.132		21		–	–	–
UBE2C Exp	Low expression	91	29	< 0.0001*	1	–	0.004*	24	0.0008*	1	–	0.022*
	High expression	91	56		2.087	1.268–3.438		41		1.846	1.092–3.121	

^*^Statistically significant

### Gene function of *UBE2C* is correlated with G2/MI transition of meiotic cell cycle

**Fig. 4:**
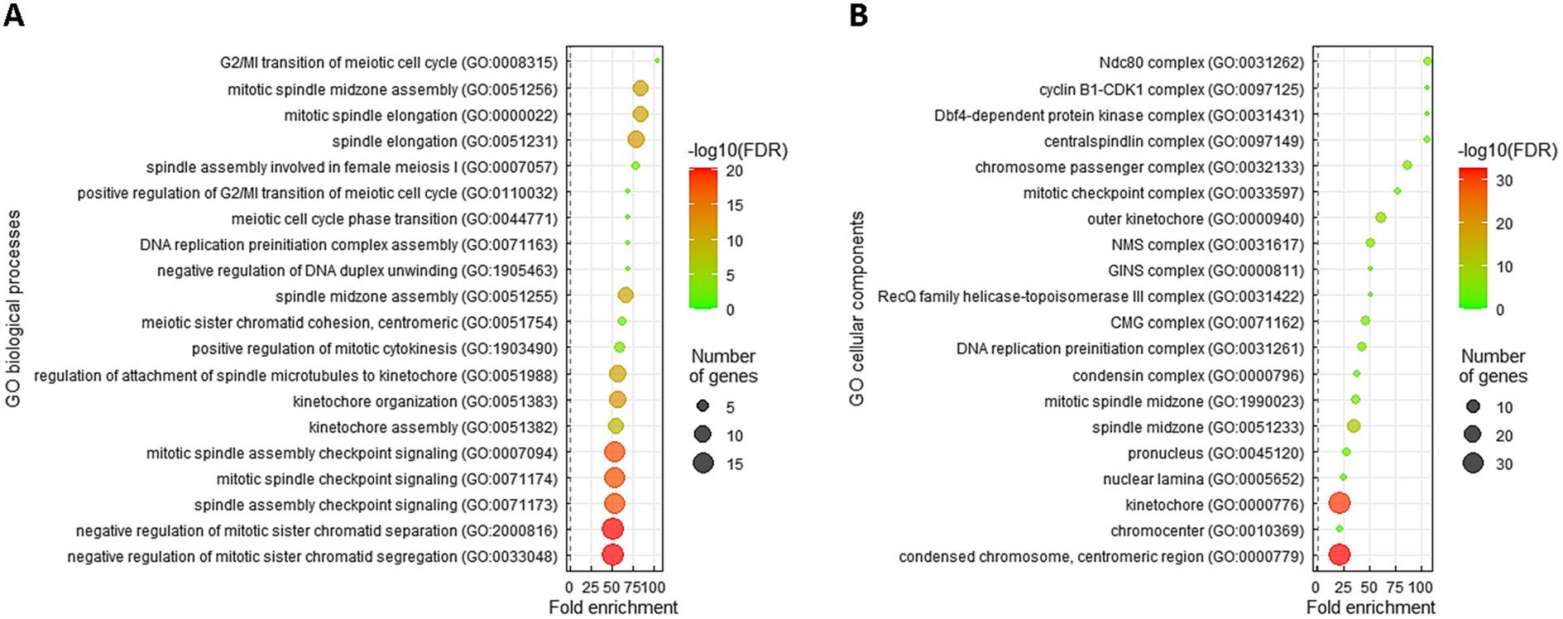
Notable GO terms enriched in UBE2C upregulation. The top 200 differentially expressed genes presenting positive connections with UBE2C were utilized to conduct functional annotation using the GO classification system according to (**A**) biological processes and (**B**) cellular components and were ordered by fold enrichment

To understand the undisclosed functions of UBE2C in IHCC, we downloaded the top 200 differentially expressed genes presenting a positive relationship (Additional file 1: Table S1) or a negative relationship (Additional file 1: Table S2) to UBE2C from the cholangiocarcinoma data set (TCGA, Firehose Legacy, *n* = 51). Subsequently, applying the Gene Ontology (GO) classification system, these genes were used to predict UBE2C functions. The results revealed that the most prominent term correlated with UBE2C upregulation was G2/MI transition of meiotic cell cycle (GO: 0008315, fold enrichment: > 100) in the context of biological processes (Fig. 4A), and the G2/mitotic-specific cyclin-B2 (*CCNB2*) and NDC80 kinetochore complex component (*NDC80*) genes were identified. In terms of cellular components, the most notable terms correlated with UBE2C upregulation were Ndc80 complex (GO: 0031262, fold enrichment: > 100) that contains the kinetochore protein Nuf2 (NUF2), kinetochore protein Spc24 (SPC24), SPC25, and NDC80 genes and cyclin B1-CDK1 complex (GO: 0097125, fold enrichment: > 100) that includes the CCNB1 and cyclin-dependent kinase 1 (CDK1) genes (Fig. 4B).

## Discussion

Cholangiocarcinoma is one of the most aggressive and lethal malignant tumors [24]. It is worth noting that, although cholangiocarcinoma accounts for a small number of all the cancers diagnosed, its incidence is on the rise globally, representing a significant global public health problem with a major impact on the individual and the social economy [25]. According to the original location of cholangiocarcinoma, it is classified into three subtypes: intrahepatic cholangiocarcinoma (about 10%), extrahepatic cholangiocarcinoma (about 50%), and distal cholangiocarcinoma (about 40%) [26]. Typically, these three cholangiocarcinomas are challenging to diagnose until they have spread to other tissues at an advanced stage [27]. Recent large-scale studies have indicated that many genes (oncogenes and tumor sup-pressors genes) have been mutations or deletions in cholangiocarcinoma, allowing cells to grow and divide uncontrollably [28, 29]. Nevertheless, it is unclear what causes the changes that lead to cholangiocarcinoma. In addition, researchers have also investigated several risk factors that may play a significant role in cholangiocarcinoma pathogenesis, including primary sclerosing cholangitis, chronic liver disease, smoking, diabetes, and a liver parasite [30, 31]. More importantly, owing to its indistinct clinical characteristics that lead to difficulties in early diagnosis and a poor prognosis, the median survival rate of cholangiocarcinoma is less than 2 years [32, 33]. To overcome the limitations of conventional treatment in cholangiocarcinoma, identifying novel biological markers that are available for the diagnostic strategies of cholangiocarcinoma is imperative.

In our study, we comparatively analyzed the differentially expressed genes from the cholangiocarcinoma transcriptome data set (GSE26566) and compared them with the gene associated with ubiquitination (GO:0016567). We validated that ubiquitination-associated gene UBE2C had the highest expression in cholangiocarcinoma patients’ tumors. UBE2C is an essential protein-coding gene in the ubiquitin-binding enzyme family, and it is involved in protein degradation, especially in cell cycle progression [34]. More importantly, many studies have indicated that UBE2C also plays an essential role in the progression of different cancer types. For example, UBE2C is highly expressed in gastric cancer, and inhibition of UBE2C expression decreases the development of gastric adenocarcinoma through the Wnt/β-catenin and PI3K/Akt signaling pathways [35]. In additionally, overexpression of UBE2C promoted cell proliferation, migration/invasion, and epithelial–mesenchymal transition (EMT) in endometrial cancer [18]. Zhenning Jin and colleagues indicated that UBE2C was upregulated in head and neck squamous cell carcinoma, and downregulation of UBE2C significantly suppressed cell migration and cell invasion [36]. Moreover, there have been multiple reports that UBE2C is an oncogene and correlates with poor survival outcomes in lung cancer [12], gastric cancer [22], hepatocellular carcinoma [37], and breast cancer [38]. However, the expression of UBE2C in survival outcomes and prognosis of cholangiocarcinoma patients is still unclear. Our study indicated that UBE2C protein expression was significantly increased in cholangiocarcinoma tumor tissues compared to non-tumor tissues by immunohistochemistry. Furthermore, we demonstrated that high expression of UBE2C was significantly correlated with poor overall survival, disease-specific survival, local recurrence-free survival, and metastasis-free survival in patients with cholangiocarcinoma. Taken together, these results suggested that UBE2C may serve as a prognostic marker of cholangiocarcinoma.

To the best of our knowledge, this study is the first to describe the association between UBE2C and the clinical characteristics of cholangiocarcinoma patients. Our clinicopathological variables indicated that UBE2C expression is significantly associated with surgical margin (R0 and R1; *p*-value = 0.029), primary tumor (T1, T2, and T3; *p*-value = 0.031), histological variant (large duct type and small duct type; *p*-value = 0.001), and histological grade (well-differentiated, moderately differentiated, and poorly differentiated; *p*-value = 0.023). More importantly, we also performed univariate and multivariate analyses to evaluate the association between the prognostic significance of UBE2C ex-pression and patients’ survival. Univariate and multivariate survival analyses showed that gender, surgical margin, primary tumor, and UBE2C expression were significantly related to overall survival, disease-specific survival, local recurrence-free survival, and metastasis-free survival. These studies suggested that UBE2C might serve as a reliable indicator for prognosis in patients with cholangiocarcinoma.

Cancer is featured by unrestrained proliferation following aberrant activity of distinct cell cycle proteins in different phases (G0/G1, S, G2, and M). In the G2/M phase of the cell cycle, CCNB1 and CCNB2 can form complexes with CDK1 to regulate the initiation of mitosis [39]. High CCNB1, CCNB2, and CDK1 expression has been associated with inferior prognosis in hepatocellular carcinoma patients [40]. In additionally, during mitotic cell division, the highly conserved NDC80 kinetochore complex creates the outer kinetochore to interact with microtubules, ensuring appropriate chromosome segregation [41]. It has also been suggested that high NDC80 complex expression is correlated with worse survival in hepatocellular carcinoma patients [42]. The NDC80 complex is composed of four components, comprising NDC80, NUF2, SPC24, and SPC25, and aberrant expression of these four components may cause uncontrolled cell proliferation in hepatocellular carcinoma [43–45]. Interestingly, we identified that the mRNA levels of CCNB1, CCNB2, CDK1, NDC80, NUF2, SPC24, and SPC25 were significantly positively correlated with UBE2C (Additional file 1: Table S1 and Fig. 4), implying that UBE2C may promote IHCC development through cell cycle regulation, and further experimental validation is needed.

Our research has certain limitations. Firstly, it is a retrospective study conducted at a single institution. Secondly, the exact molecular mechanism underlying disease progression and adverse outcomes in UBE2C-overexpressing IHCC remains unclear. Thirdly, there is currently no standardized immunostaining and scoring scheme for assessing UBEC2 expression. Lastly, to validate our findings, prospective multicenter studies are required.

## Conclusions

This study is the first to illustrate that UBE2C may be a potential marker for evaluating the prognosis of cholangiocarcinoma patients. Our cross-analysis using published transcriptome data sets and our clinical cohort demonstrated that UBE2C was significantly highly expressed in cholangiocarcinoma tumor tissues, and high expression of UBE2C was significantly associated with worse overall survival, disease-specific survival, local recurrence-free survival, and metastasis-free survival in patients with cholangiocarcinoma. Retrospective analysis determined that UBE2C expression was significantly associated with surgical margin, primary tumor, histological variant, and histological grade in cholangiocarcinoma patients. The findings from the present study may also provide a potential therapeutic marker and prognostic factor for cholangiocarcinoma patients.

### Supplementary Information

**Additional file 1: Table S1.** The top 200 genes positively correlated with UBE2C. **Table S2.** The top 200 genes negatively correlated with UBE2C.

## Data Availability

The transcriptome data set (GSE26566) analyzed in the current study is available in a published archive from the Gene Expression Omnibus (GEO) database (National Center for Biotechnology Information, Bethesda, MD, USA).
