# Supplementary material for: Ubiquitin-conjugating enzyme E2C (UBE2C) is a prognostic indicator for cholangiocarcinoma
Source: Eur J Med Res. 2023 Dec 15;28:593. doi: 10.1186/s40001-023-01575-9 (PMC10724938; doi:10.1186/s40001-023-01575-9)
Supplement: Supplementary file 1 — Additional file 1: Table S1. The top 200 genes positively correlated with UBE2C. Table S2. The top 200 genes negatively correlated with UBE2C. [file 40001_2023_1575_MOESM1_ESM.docx]

**Supplementary Table 1. The top 200 genes positively correlated with UBE2C.**

| **Correlated Gene** | **Cytoband** | **Spearman's Correlation** | **p-Value** | **q-Value** |
| --- | --- | --- | --- | --- |
| **PTTG1** | 5q33.3 | 0.877 | 2.40E-12 | **4.67E-08** |
| **PLK1** | 16p12.2 | 0.867 | 7.88E-12 | **5.60E-08** |
| **CDCA3** | 12p13.31 | 0.866 | 8.64E-12 | **5.60E-08** |
| **CCNB2** | 15q22.2 | 0.853 | 4.16E-11 | **2.02E-07** |
| **TPX2** | 20q11.21 | 0.85 | 5.46E-11 | **2.12E-07** |
| **UBE2S** | 19q13.42 | 0.837 | 2.04E-10 | **6.62E-07** |
| **BIRC5** | 17q25.3 | 0.822 | 7.83E-10 | **1.90E-06** |
| **AURKB** | 17p13.1 | 0.822 | 7.83E-10 | **1.90E-06** |
| **TROAP** | 12q13.12 | 0.819 | 1.02E-09 | **2.21E-06** |
| **ORC6** | 16q11.2 | 0.811 | 2.08E-09 | **3.75E-06** |
| **SAPCD2** | 9q34.3 | 0.809 | 2.31E-09 | **3.75E-06** |
| **CENPA** | 2p23.3 | 0.809 | 2.36E-09 | **3.75E-06** |
| **CDK1** | 10q21.2 | 0.808 | 2.51E-09 | **3.75E-06** |
| **PIF1** | 15q22.31 | 0.806 | 2.96E-09 | **4.11E-06** |
| **MYBL2** | 20q13.12 | 0.804 | 3.62E-09 | **4.49E-06** |
| **TACC3** | 4p16.3 | 0.803 | 3.85E-09 | **4.49E-06** |
| **CCNA2** | 4q27 | 0.803 | 3.93E-09 | **4.49E-06** |
| **SPC25** | 2q24.3 | 0.802 | 4.25E-09 | **4.60E-06** |
| **MAD2L1** | 4q27 | 0.793 | 7.93E-09 | **8.12E-06** |
| **SPAG5** | 17q11.2 | 0.787 | 1.29E-08 | **1.25E-05** |
| **DLGAP5** | 14q22.3 | 0.784 | 1.58E-08 | **1.46E-05** |
| **CDCA5** | 11q13.1 | 0.781 | 1.92E-08 | **1.65E-05** |
| **CDC20** | 1p34.2 | 0.781 | 1.95E-08 | **1.65E-05** |
| **GTSE1** | 22q13.31 | 0.775 | 2.83E-08 | **2.29E-05** |
| **CDCA8** | 1p34.3 | 0.77 | 3.98E-08 | **3.09E-05** |
| **PIMREG** | 17p13.2 | 0.768 | 4.62E-08 | **3.44E-05** |
| **CCNB1** | 5q13.2 | 0.767 | 4.78E-08 | **3.44E-05** |
| **SKA1** | 18q21.1 | 0.766 | 5.11E-08 | **3.48E-05** |
| **PBK** | 8p21.1 | 0.766 | 5.19E-08 | **3.48E-05** |
| **FANCA** | 16q24.3 | 0.765 | 5.64E-08 | **3.66E-05** |
| **KIF23** | 15q23 | 0.764 | 6.02E-08 | **3.78E-05** |
| **AURKA** | 20q13.2 | 0.763 | 6.32E-08 | **3.84E-05** |
| **FOXM1** | 12p13.33 | 0.761 | 6.97E-08 | **4.11E-05** |
| **FANCI** | 15q26.1 | 0.76 | 7.68E-08 | **4.39E-05** |
| **RECQL4** | 8q24.3 | 0.754 | 1.07E-07 | **5.95E-05** |
| **KIF18B** | 17q21.31 | 0.753 | 1.14E-07 | **6.16E-05** |
| **SKA3** | 13q12.11 | 0.751 | 1.29E-07 | **6.74E-05** |
| **AUNIP** | 1p36.11 | 0.751 | 1.35E-07 | **6.74E-05** |
| **EZH2** | 7q36.1 | 0.751 | 1.35E-07 | **6.74E-05** |
| **NDC80** | 18p11.32 | 0.749 | 1.46E-07 | **7.09E-05** |
| **HJURP** | 2q37.1 | 0.747 | 1.62E-07 | **7.69E-05** |
| **CDC45** | 22q11.21 | 0.747 | 1.70E-07 | **7.86E-05** |
| **POC1A** | 3p21.2 | 0.746 | 1.80E-07 | **8.15E-05** |
| **NUF2** | 1q23.3 | 0.743 | 2.16E-07 | **9.53E-05** |
| **KIFC1** | 6p21.32 | 0.741 | 2.36E-07 | **1.02E-04** |
| **CEP55** | 10q23.33 | 0.741 | 2.43E-07 | **1.03E-04** |
| **CENPW** | 6q22.32 | 0.737 | 2.97E-07 | **1.23E-04** |
| **CDC25C** | 5q31.2 | 0.735 | 3.38E-07 | **1.37E-04** |
| **STMN1** | 1p36.11 | 0.734 | 3.48E-07 | **1.38E-04** |
| **POLQ** | 3q13.33 | 0.733 | 3.79E-07 | **1.48E-04** |
| **PKMYT1** | 16p13.3 | 0.732 | 3.96E-07 | **1.51E-04** |
| **TOP2A** | 17q21.2 | 0.73 | 4.43E-07 | **1.66E-04** |
| **CKS2** | 9q22.2 | 0.729 | 4.55E-07 | **1.67E-04** |
| **FAM72B** | 1p11.2 | 0.729 | 4.68E-07 | **1.69E-04** |
| **SHCBP1** | 16q11.2 | 0.726 | 5.52E-07 | **1.95E-04** |
| **RAD51** | 15q15.1 | 0.725 | 5.68E-07 | **1.97E-04** |
| **PRC1** | 15q26.1 | 0.724 | 6.16E-07 | **2.10E-04** |
| **KIF2C** | 1p34.1 | 0.723 | 6.42E-07 | **2.15E-04** |
| **ASF1B** | 19p13.12 | 0.721 | 6.87E-07 | **2.26E-04** |
| **BUB1** | 2q13 | 0.72 | 7.25E-07 | **2.35E-04** |
| **ERCC6L** | Xq13.1 | 0.72 | 7.55E-07 | **2.41E-04** |
| **RACGAP1** | 12q13.12 | 0.717 | 8.85E-07 | **2.78E-04** |
| **KIF4A** | Xq13.1 | 0.714 | 1.02E-06 | **3.15E-04** |
| **TICRR** | 15q26.1 | 0.714 | 1.04E-06 | **3.15E-04** |
| **KIF4B** | 5q33.2 | 0.712 | 1.12E-06 | **3.35E-04** |
| **DEPDC1** | 1p31.3 | 0.71 | 1.21E-06 | **3.57E-04** |
| **FAM83D** | 20q11.23 | 0.71 | 1.24E-06 | **3.61E-04** |
| **LMNB1** | 5q23.2 | 0.709 | 1.29E-06 | **3.63E-04** |
| **KIF15** | 3p21.31 | 0.709 | 1.32E-06 | **3.63E-04** |
| **RAD51AP1** | 12p13.32 | 0.709 | 1.32E-06 | **3.63E-04** |
| **WDR62** | 19q13.12 | 0.708 | 1.36E-06 | **3.67E-04** |
| **BUB1B** | 15q15.1 | 0.706 | 1.49E-06 | **3.96E-04** |
| **MND1** | 4q31.3 | 0.698 | 2.19E-06 | **5.67E-04** |
| **CENPE** | 4q24 | 0.698 | 2.19E-06 | **5.67E-04** |
| **ZWINT** | 10q21.1 | 0.696 | 2.41E-06 | **6.17E-04** |
| **RNASEH2A** | 19p13.13 | 0.695 | 2.50E-06 | **6.31E-04** |
| **BLM** | 15q26.1 | 0.695 | 2.53E-06 | **6.31E-04** |
| **DDIAS** | 11q14.1 | 0.694 | 2.69E-06 | **6.61E-04** |
| **ARHGAP11A** | 15q13.3 | 0.692 | 2.96E-06 | **7.19E-04** |
| **NCAPG** | 4p15.31 | 0.691 | 3.07E-06 | **7.36E-04** |
| **ANLN** | 7p14.2 | 0.69 | 3.22E-06 | **7.62E-04** |
| **CDC25A** | 3p21.31 | 0.686 | 3.79E-06 | **8.78E-04** |
| **SGO1** | 3p24.3 | 0.686 | 3.79E-06 | **8.78E-04** |
| **PHF19** | 9q33.2 | 0.683 | 4.46E-06 | **1.02E-03** |
| **RACGAP1P** | 12q12 | 0.682 | 4.60E-06 | **1.03E-03** |
| **RAD54L** | 1p34.1 | 0.682 | 4.62E-06 | **1.03E-03** |
| **DBF4** | 7q21.12 | 0.68 | 5.00E-06 | **1.11E-03** |
| **LRR1** | 14q21.3 | 0.671 | 7.49E-06 | **1.64E-03** |
| **HMGB2** | 4q34.1 | 0.671 | 7.58E-06 | **1.64E-03** |
| **MELK** | 9p13.2 | 0.67 | 7.75E-06 | **1.66E-03** |
| **E2F1** | 20q11.22 | 0.67 | 7.92E-06 | **1.67E-03** |
| **TTK** | 6q14.1 | 0.668 | 8.46E-06 | **1.77E-03** |
| **KNSTRN** | 15q15.1 | 0.664 | 1.01E-05 | **2.08E-03** |
| **NEK2** | 1q32.3 | 0.662 | 1.12E-05 | **2.27E-03** |
| **ZNHIT3** | 17q12 | 0.662 | 1.12E-05 | **2.27E-03** |
| **HMMR** | 5q34 | 0.66 | 1.21E-05 | **2.42E-03** |
| **ESPL1** | 12q13.13 | 0.659 | 1.22E-05 | **2.42E-03** |
| **UBE2T** | 1q32.1 | 0.658 | 1.28E-05 | **2.52E-03** |
| **PSMD3** | 17q21.1 | 0.656 | 1.40E-05 | **2.71E-03** |
| **KIF11** | 10q23.33 | 0.655 | 1.47E-05 | **2.83E-03** |
| **PARPBP** | 12q23.2 | 0.654 | 1.53E-05 | **2.89E-03** |
| **CIT** | 12q24.23 | 0.654 | 1.53E-05 | **2.89E-03** |
| **FAM72A** | 1q32.1 | 0.652 | 1.63E-05 | **3.05E-03** |
| **TK1** | 17q25.3 | 0.652 | 1.65E-05 | **3.05E-03** |
| **LMNB2** | 19p13.3 | 0.651 | 1.72E-05 | **3.15E-03** |
| **OSER1** | 20q13.12 | 0.645 | 2.15E-05 | **3.90E-03** |
| **E2F8** | 11p15.1 | 0.638 | 2.89E-05 | **5.20E-03** |
| **MKI67** | 10q26.2 | 0.632 | 3.60E-05 | **6.37E-03** |
| **CCNF** | 16p13.3 | 0.631 | 3.71E-05 | **6.50E-03** |
| **MTFR2** | 6q23.3 | 0.63 | 3.78E-05 | **6.56E-03** |
| **EME1** | 17q21.33 | 0.63 | 3.85E-05 | **6.63E-03** |
| **TRIP13** | 5p15.33 | 0.627 | 4.28E-05 | **7.29E-03** |
| **KIF20A** | 5q31.2 | 0.625 | 4.52E-05 | **7.58E-03** |
| **NCAPD2** | 12p13.31 | 0.624 | 4.74E-05 | **7.88E-03** |
| **MCM2** | 3q21.3 | 0.624 | 4.78E-05 | **7.88E-03** |
| **DTYMK** | 2q37.3 | 0.622 | 5.15E-05 | **8.42E-03** |
| **RCC1** | 1p35.3 | 0.62 | 5.44E-05 | **8.82E-03** |
| **GTSE1-DT** | 22q13.31 | 0.62 | 5.49E-05 | **8.83E-03** |
| **E2F2** | 1p36.12 | 0.62 | 5.60E-05 | **8.92E-03** |
| **PCNA** | 20p12.3 | 0.619 | 5.75E-05 | **9.09E-03** |
| **CEP78** | 9q21.2 | 0.617 | 6.08E-05 | **9.53E-03** |
| **GINS2** | 16q24.1 | 0.617 | 6.19E-05 | **9.62E-03** |
| **NEIL3** | 4q34.3 | 0.615 | 6.47E-05 | **9.91E-03** |
| **AGAP3** | 7q36.1 | 0.615 | 6.47E-05 | **9.91E-03** |
| **RDM1** | 17q12 | 0.612 | 7.28E-05 | **0.0111** |
| **CENPF** | 1q41 | 0.612 | 7.34E-05 | **0.0111** |
| **DBF4B** | 17q21.31\|17q21 | 0.611 | 7.47E-05 | **0.0112** |
| **DIAPH3** | 13q21.2 | 0.608 | 8.24E-05 | **0.0122** |
| **FAM72D** | 1q21.1 | 0.605 | 9.15E-05 | **0.0134** |
| **SP5** | 2q31.1 | 0.605 | 9.15E-05 | **0.0134** |
| **SPC24** | 19p13.2 | 0.605 | 9.23E-05 | **0.0134** |
| **IQGAP3** | 1q22 | 0.604 | 9.73E-05 | **0.014** |
| **CENPK** | 5q12.3 | 0.603 | 1.01E-04 | **0.0144** |
| **KIF18A** | 11p14.1 | 0.602 | 1.02E-04 | **0.0144** |
| **CCNE1** | 19q12 | 0.601 | 1.05E-04 | **0.0148** |
| **NCAPG2** | 7q36.3 | 0.601 | 1.08E-04 | **0.0151** |
| **PTTG3P** | 8q13.1 | 0.596 | 1.24E-04 | **0.0172** |
| **ACSF2** | 17q21.33 | 0.595 | 1.31E-04 | **0.018** |
| **RCCD1** | 15q26.1 | 0.594 | 1.34E-04 | **0.018** |
| **HASPIN** | 17p13.2 | 0.594 | 1.34E-04 | **0.018** |
| **EXO1** | 1q43 | 0.591 | 1.46E-04 | **0.0196** |
| **CENPH** | 5q13.2 | 0.59 | 1.51E-04 | **0.0202** |
| **CDCA2** | 8p21.2 | 0.59 | 1.53E-04 | **0.0202** |
| **CDKN2C** | 1p32.3 | 0.587 | 1.66E-04 | **0.0218** |
| **THOP1** | 19p13.3 | 0.587 | 1.67E-04 | **0.0218** |
| **PLK4** | 4q28.1 | 0.587 | 1.69E-04 | **0.0218** |
| **KIF22** | 16p11.2 | 0.585 | 1.78E-04 | **0.023** |
| **RMI2** | 16p13.13 | 0.585 | 1.80E-04 | **0.023** |
| **RFC4** | 3q27.3 | 0.585 | 1.81E-04 | **0.023** |
| **RNFT2** | 12q24.22 | 0.584 | 1.84E-04 | **0.0233** |
| **MVD** | 16q24.2 | 0.579 | 2.15E-04 | **0.027** |
| **INCENP** | 11q12.3 | 0.578 | 2.24E-04 | **0.0277** |
| **TCF19** | 6p21.33 | 0.577 | 2.33E-04 | **0.0286** |
| **SNRPA1** | 15q26.3 | 0.575 | 2.42E-04 | **0.0296** |
| **TEDC2** | 16p13.3 | 0.575 | 2.44E-04 | **0.0297** |
| **TMPO-AS1** | 12q23.1 | 0.575 | 2.48E-04 | **0.0298** |
| **PCLAF** | 15q22.31 | 0.575 | 2.48E-04 | **0.0298** |
| **H2AFX** | 11q23.3 | 0.574 | 2.52E-04 | **0.03** |
| **KPNA2** | 17q24.2 | 0.573 | 2.60E-04 | **0.0308** |
| **FANCD2** | 3p25.3 | 0.572 | 2.68E-04 | **0.0316** |
| **GSDMB** | 17q21.1 | 0.572 | 2.70E-04 | **0.0317** |
| **SIX1** | 14q23.1 | 0.571 | 2.76E-04 | **0.0321** |
| **RRM2** | 2p25.1 | 0.571 | 2.78E-04 | **0.0321** |
| **HELLS** | 10q23.33 | 0.569 | 2.90E-04 | **0.0332** |
| **ATAD3A** | 1p36.33 | 0.569 | 2.92E-04 | **0.0332** |
| **SGO2** | 2q33.1 | 0.568 | 3.01E-04 | **0.0337** |
| **ECT2** | 3q26.31 | 0.568 | 3.01E-04 | **0.0337** |
| **CENPU** | 4q35.1 | 0.567 | 3.09E-04 | **0.0343** |
| **WRAP73** | 1p36.32 | 0.563 | 3.46E-04 | **0.0382** |
| **REXO5** | 16p12.3 | 0.561 | 3.73E-04 | **0.041** |
| **CENPN** | 16q23.2 | 0.56 | 3.79E-04 | **0.0414** |
| **MYB** | 6q23.3 | 0.558 | 4.03E-04 | **0.0435** |
| **USP39** | 2p11.2 | 0.557 | 4.21E-04 | **0.045** |
| **RANBP1** | 22q11.21 | 0.554 | 4.60E-04 | **0.0489** |
| **NOC2L** | 1p36.33 | 0.551 | 4.99E-04 | 0.0527 |
| **ASPM** | 1q31.3 | 0.551 | 5.03E-04 | 0.0528 |
| **RHNO1** | 12p13.33 | 0.546 | 5.77E-04 | 0.0594 |
| **ATAD3B** | 1p36.33 | 0.546 | 5.77E-04 | 0.0594 |
| **SLC26A6** | 3p21.31 | 0.545 | 5.85E-04 | 0.0599 |
| **C18ORF54** | 18q21.2 | 0.545 | 5.90E-04 | 0.06 |
| **KIF14** | 1q32.1 | 0.545 | 5.94E-04 | 0.0601 |
| **CDKN2D** | 19p13.2 | 0.543 | 6.16E-04 | 0.062 |
| **DUS1L** | 17q25.3 | 0.543 | 6.24E-04 | 0.0623 |
| **CDC6** | 17q21.2 | 0.543 | 6.24E-04 | 0.0623 |
| **ZNF593** | 1p36.11 | 0.541 | 6.52E-04 | 0.0645 |
| **CIP2A** | 3q13.13 | 0.541 | 6.56E-04 | 0.0645 |
| **CDKN3** | 14q22.2 | 0.541 | 6.56E-04 | 0.0645 |
| **H2BFXP** | Xq22.2 | 0.54 | 6.70E-04 | 0.0652 |
| **KLHL17** | 1p36.33 | 0.54 | 6.70E-04 | 0.0652 |
| **MCM7** | 7q22.1 | 0.539 | 6.99E-04 | 0.0677 |
| **ARHGEF39** | 9p13.3 | 0.538 | 7.09E-04 | 0.0683 |
| **PSMD7** | 16q23.1 | 0.538 | 7.14E-04 | 0.0684 |
| **GINS1** | 20p11.21 | 0.538 | 7.19E-04 | 0.0686 |
| **MPHOSPH6** | 16q23.3 | 0.537 | 7.35E-04 | 0.0697 |
| **CMSS1** | 3q12.1 | 0.535 | 7.77E-04 | 0.0733 |
| **FBXO43** | 8q22.2 | 0.534 | 7.93E-04 | 0.0734 |
| **TUBA1B** | 12q13.12 | 0.534 | 7.99E-04 | 0.0734 |
| **CDT1** | 16q24.3 | 0.534 | 8.04E-04 | 0.0734 |
| **MME** | 3q25.2 | 0.534 | 8.04E-04 | 0.0734 |
| **BET1** | 7q21.3 | 0.533 | 8.21E-04 | 0.0746 |

**Supplementary Table 2. The top 200 genes negatively correlated with UBE2C.**

| **Correlated Gene** | **Cytoband** | **Spearman's Correlation** | **p-Value** | **q-Value** |
| --- | --- | --- | --- | --- |
| **KCND3** | 1p13.2 | -0.709 | 1.27E-06 | **3.63E-04** |
| **TNKS1BP1** | 11q12.1 | -0.636 | 3.06E-05 | **5.46E-03** |
| **FAM161B** | 14q24.3 | -0.625 | 4.52E-05 | **7.58E-03** |
| **ZNF43** | 19p12 | -0.596 | 1.26E-04 | **0.0174** |
| **ZNF737** | 19p12 | -0.578 | 2.24E-04 | **0.0277** |
| **MARF1** | 16p13.11 | -0.57 | 2.81E-04 | **0.0323** |
| **MTREX** | 5q11.2 | -0.568 | 2.99E-04 | **0.0337** |
| **SLC17A1** | 6p22.2 | -0.559 | 3.92E-04 | **0.0425** |
| **C11ORF52** | 11q23.1 | -0.557 | 4.18E-04 | **0.0449** |
| **C11ORF54** | 11q21 | -0.549 | 5.29E-04 | 0.055 |
| **PPFIBP1** | 12p11.23-p11.22 | -0.549 | 5.29E-04 | 0.055 |
| **FAXDC2** | 5q33.2 | -0.534 | 7.88E-04 | 0.0734 |
| **ZNF429** | 19p12 | -0.534 | 7.93E-04 | 0.0734 |
| **ZBTB20** | 3q13.31 | -0.534 | 7.99E-04 | 0.0734 |
| **ZNF641** | 12q13.11 | -0.529 | 8.98E-04 | 0.0798 |
| **ZBTB4** | 17p13.1 | -0.528 | 9.23E-04 | 0.0809 |
| **FCHO2** | 5q13.2 | -0.525 | 1.00E-03 | 0.0866 |
| **EPC1** | 10p11.22 | -0.525 | 1.02E-03 | 0.0874 |
| **CLCC1** | 1p13.3 | -0.524 | 1.05E-03 | 0.0899 |
| **PCDHB3** | 5q31.3 | -0.523 | 1.07E-03 | 0.091 |
| **CHM** | Xq21.2 | -0.52 | 1.15E-03 | 0.0944 |
| **ARHGEF12** | 11q23.3 | -0.52 | 1.15E-03 | 0.0944 |
| **STUM** | 1q42.12 | -0.518 | 1.22E-03 | 0.0988 |
| **CC2D2A** | 4p15.32 | -0.516 | 1.27E-03 | 0.101 |
| **SFT2D2** | 1q24.2 | -0.516 | 1.27E-03 | 0.101 |
| **IL6ST** | 5q11.2 | -0.515 | 1.33E-03 | 0.103 |
| **POLK** | 5q13.3 | -0.514 | 1.33E-03 | 0.104 |
| **DIXDC1** | 11q23.1 | -0.514 | 1.36E-03 | 0.105 |
| **ARHGEF17** | 11q13.4 | -0.512 | 1.40E-03 | 0.106 |
| **CUL5** | 11q22.3 | -0.511 | 1.43E-03 | 0.109 |
| **HBG2** | 11p15.4 | -0.511 | 1.46E-03 | 0.11 |
| **ABCD3** | 1p21.3 | -0.509 | 1.51E-03 | 0.113 |
| **RTN4RL1** | 17p13.3 | -0.509 | 1.53E-03 | 0.114 |
| **CDKL1** | 14q21.3 | -0.508 | 1.58E-03 | 0.117 |
| **CDSN** | 6p21.33 | -0.506 | 1.63E-03 | 0.12 |
| **PRR26** | 10p15.3 | -0.506 | 1.65E-03 | 0.121 |
| **FPGT** | 1p31.1 | -0.503 | 1.75E-03 | 0.127 |
| **DYDC2** | 10q23.1 | -0.502 | 1.82E-03 | 0.13 |
| **BLOC1S5** | 6p24.3 | -0.501 | 1.84E-03 | 0.13 |
| **PARD3** | 10p11.22-p11.21 | -0.501 | 1.85E-03 | 0.13 |
| **DYDC1** | 10q23.1 | -0.499 | 1.95E-03 | 0.134 |
| **ZBTB44** | 11q24.3 | -0.499 | 1.97E-03 | 0.134 |
| **MMAA** | 4q31.21 | -0.498 | 1.98E-03 | 0.135 |
| **LINC01278** | Xq11.1 | -0.498 | 2.02E-03 | 0.135 |
| **ZNF253** | 19p13.11 | -0.494 | 2.21E-03 | 0.146 |
| **DHX29** | 5q11.2 | -0.491 | 2.34E-03 | 0.152 |
| **PTPRJ** | 11p11.2 | -0.491 | 2.38E-03 | 0.154 |
| **CTNND1** | 11q12.1 | -0.49 | 2.40E-03 | 0.154 |
| **SLC16A7** | 12q14.1 | -0.49 | 2.40E-03 | 0.154 |
| **EPHA3** | 3p11.1 | -0.488 | 2.55E-03 | 0.163 |
| **TUBBP6** | 7p11.2 | -0.486 | 2.65E-03 | 0.168 |
| **GRID1** | 10q23.1-q23.2 | -0.485 | 2.71E-03 | 0.168 |
| **SHC1** | 1q21.3 | -0.485 | 2.71E-03 | 0.168 |
| **FBXO3** | 11p13 | -0.485 | 2.74E-03 | 0.17 |
| **BAZ2B** | 2q24.2 | -0.483 | 2.82E-03 | 0.172 |
| **HIST1H2BC** | 6p22.2 | -0.483 | 2.82E-03 | 0.172 |
| **GCNT4** | 5q13.3 | -0.483 | 2.82E-03 | 0.172 |
| **FBXL17** | 5q21.3 | -0.483 | 2.86E-03 | 0.173 |
| **HMBOX1** | 8p21.1-p12 | -0.481 | 2.98E-03 | 0.179 |
| **TRAPPC13** | 5q12.3 | -0.481 | 2.99E-03 | 0.179 |
| **AFF4** | 5q31.1 | -0.481 | 2.99E-03 | 0.179 |
| **OCLN** | 5q13.2 | -0.48 | 3.05E-03 | 0.181 |
| **MFSD14A** | 1p21.2 | -0.478 | 3.18E-03 | 0.186 |
| **ZNF616** | 19q13.41 | -0.478 | 3.21E-03 | 0.188 |
| **ADAMTS8** | 11q24.3 | -0.477 | 3.27E-03 | 0.189 |
| **PLXDC2** | 10p12.31 | -0.475 | 3.39E-03 | 0.195 |
| **KCTD20** | 6p21.31 | -0.475 | 3.41E-03 | 0.195 |
| **SPSB1** | 1p36.22 | -0.475 | 3.45E-03 | 0.196 |
| **CUL4B** | Xq24 | -0.474 | 3.47E-03 | 0.196 |
| **FGGY** | 1p32.1 | -0.473 | 3.55E-03 | 0.198 |
| **BCLAF3** | Xp22.12 | -0.473 | 3.59E-03 | 0.199 |
| **TRIP11** | 14q32.12 | -0.473 | 3.61E-03 | 0.199 |
| **COL4A3BP** | 5q13.3 | -0.472 | 3.63E-03 | 0.2 |
| **PAFAH1B2** | 11q23.3 | -0.472 | 3.65E-03 | 0.201 |
| **PTPRG** | 3p14.2 | -0.472 | 3.67E-03 | 0.201 |
| **ARHGAP1** | 11p11.2 | -0.472 | 3.69E-03 | 0.201 |
| **KLHL9** | 9p21.3 | -0.471 | 3.72E-03 | 0.201 |
| **CACNA1H** | 16p13.3 | -0.47 | 3.82E-03 | 0.206 |
| **DST** | 6p12.1 | -0.469 | 3.87E-03 | 0.207 |
| **ZNF689** | 16p11.2 | -0.469 | 3.87E-03 | 0.207 |
| **ELFN1** | 7p22.3 | -0.469 | 3.91E-03 | 0.207 |
| **ANKEF1** | 20p12.2 | -0.469 | 3.93E-03 | 0.207 |
| **FNIP1** | 5q31.1 | -0.468 | 4.00E-03 | 0.21 |
| **FEM1C** | 5q22.3 | -0.464 | 4.36E-03 | 0.222 |
| **MAP9** | 4q32.1 | -0.464 | 4.41E-03 | 0.223 |
| **IFNAR1** | 21q22.11 | -0.463 | 4.48E-03 | 0.225 |
| **ATRX** | Xq21.1 | -0.462 | 4.53E-03 | 0.225 |
| **ZNF254** | 19p12 | -0.462 | 4.58E-03 | 0.226 |
| **NEXN** | 1p31.1 | -0.462 | 4.61E-03 | 0.226 |
| **SECISBP2L** | 15q21.1 | -0.461 | 4.63E-03 | 0.226 |
| **PCDHB8** | 5q31.3 | -0.461 | 4.63E-03 | 0.226 |
| **JMJD1C** | 10q21.3 | -0.461 | 4.66E-03 | 0.226 |
| **USP9X** | Xp11.4 | -0.461 | 4.69E-03 | 0.226 |
| **POF1B** | Xq21.1 | -0.46 | 4.71E-03 | 0.226 |
| **PCDHB11** | 5q31.3 | -0.459 | 4.82E-03 | 0.23 |
| **SLC17A3** | 6p22.2 | -0.459 | 4.84E-03 | 0.23 |
| **DSP** | 6p24.3 | -0.459 | 4.87E-03 | 0.23 |
| **MAP3K2** | 2q14.3 | -0.458 | 4.93E-03 | 0.232 |
| **UEVLD** | 11p15.1 | -0.457 | 5.09E-03 | 0.235 |
| **NRXN3** | 14q24.3-q31.1 | -0.457 | 5.09E-03 | 0.235 |
| **EGFR** | 7p11.2 | -0.457 | 5.12E-03 | 0.235 |
| **DUSP22** | 6p25.3 | -0.456 | 5.18E-03 | 0.235 |
| **HIPK3** | 11p13 | -0.456 | 5.18E-03 | 0.235 |
| **PKHD1** | 6p12.3-p12.2 | -0.456 | 5.18E-03 | 0.235 |
| **TRIM23** | 5q12.3 | -0.456 | 5.21E-03 | 0.235 |
| **RRAGB** | Xp11.21 | -0.456 | 5.23E-03 | 0.236 |
| **PRICKLE1** | 12q12 | -0.455 | 5.26E-03 | 0.236 |
| **TRIM33** | 1p13.2 | -0.455 | 5.35E-03 | 0.237 |
| **CPNE8** | 12q12 | -0.454 | 5.38E-03 | 0.237 |
| **ID2B** | 3p14.2 | -0.454 | 5.41E-03 | 0.238 |
| **KCNB1** | 20q13.13 | -0.452 | 5.60E-03 | 0.243 |
| **CXADR** | 21q21.1 | -0.451 | 5.71E-03 | 0.245 |
| **EFHD1** | 2q37.1 | -0.451 | 5.77E-03 | 0.247 |
| **CRY2** | 11p11.2 | -0.451 | 5.77E-03 | 0.247 |
| **PBLD** | 10q21.3 | -0.45 | 5.84E-03 | 0.248 |
| **CSNK2A3** | 11p15.4 | -0.45 | 5.84E-03 | 0.248 |
| **CGNL1** | 15q21.3 | -0.45 | 5.90E-03 | 0.248 |
| **ZBED3** | 5q13.3 | -0.45 | 5.90E-03 | 0.248 |
| **PGAP2** | 11p15.4 | -0.45 | 5.93E-03 | 0.248 |
| **PHF21A** | 11p11.2 | -0.449 | 6.00E-03 | 0.249 |
| **ATF7** | 12q13.13 | -0.449 | 6.00E-03 | 0.249 |
| **PRSS35** | 6q14.2 | -0.449 | 6.03E-03 | 0.249 |
| **PHLDB2** | 3q13.2 | -0.449 | 6.03E-03 | 0.249 |
| **CHURC1** | 14q23.3 | -0.448 | 6.13E-03 | 0.252 |
| **ERN1** | 17q23.3 | -0.447 | 6.26E-03 | 0.255 |
| **LYSMD3** | 5q14.3 | -0.447 | 6.33E-03 | 0.256 |
| **ZNF154** | 19q13.43 | -0.446 | 6.36E-03 | 0.256 |
| **THAP1** | 8p11.21 | -0.446 | 6.36E-03 | 0.256 |
| **SLC25A46** | 5q22.1 | -0.445 | 6.50E-03 | 0.259 |
| **AFF1** | 4q21.3-q22.1 | -0.445 | 6.53E-03 | 0.26 |
| **SRCAP** | 16p11.2 | -0.444 | 6.67E-03 | 0.262 |
| **SLC39A14** | 8p21.3 | -0.444 | 6.67E-03 | 0.262 |
| **NBEAL1** | 2q33.2 | -0.444 | 6.74E-03 | 0.264 |
| **ADAMTS15** | 11q24.3 | -0.443 | 6.82E-03 | 0.264 |
| **CRYAB** | 11q23.1 | -0.443 | 6.82E-03 | 0.264 |
| **CFAP126** | 1q23.3 | -0.443 | 6.85E-03 | 0.264 |
| **RAD50** | 5q31.1 | -0.443 | 6.85E-03 | 0.264 |
| **ATP1B2** | 17p13.1 | -0.442 | 6.89E-03 | 0.265 |
| **GHR** | 5p13.1-p12 | -0.441 | 7.04E-03 | 0.267 |
| **LINC00924** | 15q26.2 | -0.441 | 7.04E-03 | 0.267 |
| **PCDHB13** | 5q31.3 | -0.44 | 7.19E-03 | 0.27 |
| **CCDC153** | 11q23.3 | -0.44 | 7.19E-03 | 0.27 |
| **SCN4B** | 11q23.3 | -0.44 | 7.26E-03 | 0.27 |
| **PJA2** | 5q21.3 | -0.439 | 7.34E-03 | 0.272 |
| **PROX1** | 1q32.3 | -0.439 | 7.38E-03 | 0.273 |
| **HIST1H2BA** | 6p22.2 | -0.439 | 7.41E-03 | 0.274 |
| **MBD5** | 2q23.1 | -0.439 | 7.41E-03 | 0.274 |
| **RASAL2** | 1q25.2 | -0.438 | 7.49E-03 | 0.275 |
| **AVPR1B** | 1q32.1 | -0.438 | 7.53E-03 | 0.275 |
| **HEYL** | 1p34.2 | -0.438 | 7.53E-03 | 0.275 |
| **SYNJ1** | 21q22.11 | -0.438 | 7.57E-03 | 0.276 |
| **CYP2C18** | 10q23.33 | -0.437 | 7.65E-03 | 0.277 |
| **CCKBR** | 11p15.4 | -0.437 | 7.66E-03 | 0.277 |
| **ARHGEF40** | 14q11.2 | -0.437 | 7.73E-03 | 0.277 |
| **OGN** | 9q22.31 | -0.436 | 7.83E-03 | 0.278 |
| **SGPP1** | 14q23.2 | -0.436 | 7.93E-03 | 0.279 |
| **KATNAL1** | 13q12.3 | -0.436 | 7.93E-03 | 0.279 |
| **PIK3AP1** | 10q24.1 | -0.436 | 7.93E-03 | 0.279 |
| **ZNF426** | 19p13.2 | -0.435 | 8.02E-03 | 0.28 |
| **BPTF** | 17q24.2 | -0.435 | 8.06E-03 | 0.281 |
| **BTBD8** | 1p22.1 | -0.434 | 8.17E-03 | 0.282 |
| **FBXW11** | 5q35.1 | -0.433 | 8.27E-03 | 0.284 |
| **ATF7IP** | 12p13.1 | -0.433 | 8.27E-03 | 0.284 |
| **SBF2** | 11p15.4 | -0.433 | 8.35E-03 | 0.284 |
| **PDCD4** | 10q25.2 | -0.433 | 8.35E-03 | 0.284 |
| **RSF1** | 11q14.1 | -0.432 | 8.48E-03 | 0.285 |
| **SMIM10L2B** | Xq26.3 | -0.431 | 8.61E-03 | 0.287 |
| **FGD4** | 12p11.21 | -0.431 | 8.61E-03 | 0.287 |
| **CHD9** | 16q12.2 | -0.431 | 8.70E-03 | 0.289 |
| **LEPROT** | 1p31.3 | -0.43 | 8.79E-03 | 0.29 |
| **SLC35F5** | 2q14.1 | -0.43 | 8.84E-03 | 0.29 |
| **ZCCHC24** | 10q22.3 | -0.43 | 8.84E-03 | 0.29 |
| **BMPR2** | 2q33.1-q33.2 | -0.43 | 8.88E-03 | 0.29 |
| **PPP1R1A** | 12q13.2 | -0.43 | 8.93E-03 | 0.29 |
| **USP32** | 17q23.1-q23.2 | -0.429 | 8.97E-03 | 0.29 |
| **ZNF432** | 19q13.41 | -0.429 | 9.02E-03 | 0.291 |
| **WDFY3** | 4q21.23 | -0.428 | 9.16E-03 | 0.294 |
| **RGS7BP** | 5q12.3 | -0.428 | 9.20E-03 | 0.295 |
| **TEAD1** | 11p15.3 | -0.428 | 9.30E-03 | 0.297 |
| **ADCYAP1R1** | 7p14.3 | -0.427 | 9.31E-03 | 0.297 |
| **PARP8** | 5q11.1 | -0.427 | 9.39E-03 | 0.298 |
| **LRRC8E** | 19p13.2 | -0.426 | 9.54E-03 | 0.3 |
| **SNORC** | 2q37.1 | -0.425 | 9.68E-03 | 0.303 |
| **TNS1** | 2q35 | -0.424 | 9.88E-03 | 0.306 |
| **SBNO1** | 12q24.31 | -0.424 | 9.88E-03 | 0.306 |
| **RAB18** | 10p12.1 | -0.424 | 9.93E-03 | 0.306 |
| **CREBL2** | 12p13.1 | -0.424 | 0.01 | 0.307 |
| **BAAT** | 9q31.1 | -0.424 | 0.01 | 0.307 |
| **FER** | 5q21.3 | -0.423 | 0.0101 | 0.307 |
| **USP25** | 21q21.1 | -0.423 | 0.0102 | 0.309 |
| **GPRASP2** | Xq22.1 | -0.422 | 0.0103 | 0.31 |
| **NDRG2** | 14q11.2 | -0.422 | 0.0103 | 0.31 |
| **LNPEP** | 5q15 | -0.422 | 0.0104 | 0.312 |
| **LAMC3** | 9q34.12 | -0.422 | 0.0104 | 0.312 |
| **CTSO** | 4q32.1 | -0.422 | 0.0104 | 0.312 |
| **LPGAT1** | 1q32.3 | -0.422 | 0.0104 | 0.312 |
| **DCAF6** | 1q24.2 | -0.421 | 0.0105 | 0.312 |
| **EPB41L5** | 2q14.2 | -0.421 | 0.0105 | 0.312 |
| **CWF19L2** | 11q22.3 | -0.421 | 0.0105 | 0.312 |
| **CFAP221** | 2q14.2 | -0.421 | 0.0106 | 0.312 |
